# The Effect of Berberine on Polycystic Ovary Syndrome Patients with Insulin Resistance (PCOS-IR): A Meta-Analysis and Systematic Review

**DOI:** 10.1155/2018/2532935

**Published:** 2018-11-14

**Authors:** Meng-Fei Li, Xiao-Meng Zhou, Xue-Lian Li

**Affiliations:** ^1^OB/GYN Hospital, Fudan University, Shanghai, China; ^2^Shanghai Key Laboratory of Female Reproductive Endocrine-Related Diseases, Shanghai, China

## Abstract

**Purpose:**

To evaluate the effect of berberine (BBR) on polycystic ovary syndrome (PCOS) patients with insulin resistance (IR).

**Methods:**

PubMed (in English), Medline (in English), Embase (in English), CNKI (in Chinese), WanFang DATA (in Chinese), and VIP (in Chinese) were searched for randomized controlled trials in human beings with the search terms including “polycystic ovary syndrome /PCOS” and “berberine/BBR/Huang liansu (in Chinese)/ Xiao bojian (in Chinese)” till July 2018. Relevant indices were collected and analyzed by Stata 13.0.

**Results:**

A total of 9 randomized controlled trials were included. Limited data demonstrated the results as follows: No significant difference was found between berberine (BBR) and metformin (MET) on alleviating insulin resistance, improving glycolipid metabolism, or reproductive endocrine condition. MET combined with BBR was not superior to MET alone, but cyproterone acetate (CPA) combined with BBR was superior to CPA alone in improving some of the reproductive endocrine indices. The combination of BBR and Chinese herbs also showed positive effect. However there are insufficient data to make any conclusions on the effect of BBR on PCOS-IR.

**Conclusion:**

BBR showed a promising prospect in treating PCOS-IR. But its mechanisms are still unclear, and more properly designed, randomized, double-blind, placebo-controlled trials are needed to further confirm its effect and safety.

## 1. Introduction

Polycystic ovary syndrome (PCOS) is a common endocrine disorder among women of reproductive age [1] which is characterized by anovulation, amenorrhea, infertility, obesity, polycystic ovary, hirsutism, acne, etc. PCOS has been identified as a kind of metabolic syndrome and may lead to a long-term risk of diabetes and cardiovascular disease. It is generally believed that insulin resistance (IR), which is an increased risk of glucose intolerance and type 2 diabetes (T2D), is the core of PCOS [2] and there is about 50–75% of PCOS patients with different degrees of IR [3]. Insulin-sensitizing agents can improve the endocrine and clinical symptoms of PCOS patients in some way. Metformin (MET) is a widely used insulin-sensitizing agent, which was firstly administered in obese PCOS women in 1994 to reduce serum levels of insulin and androgen and regularize the cycle of menses, but some patients worry about the potential adverse effects.

Berberine (BBR), a major active component of the Chinese herbal medicines Rhizoma Coptidis, Cortex Phellodendri, and Cortex Berberidis, has been prescribed for the treatment of diarrhea, metabolic disorders, and infertility. It has been used for thousands of years in its herbal form to enhance fertility and recently as an extract combined with cyproterone acetate (CPA), clomiphene (CC), and letrozole (LET), to enhance their effectiveness [4–7]. Recently, a large body of evidence has demonstrated that BBR has a comparable activity to MET in reducing IR [8, 9]. This study aimed to assess the effects of BBR on PCOS-IR by meta-analysis and a systematic review in different aspects such as alleviating insulin resistance, improving glycolipid metabolism, reproductive endocrine condition and reproductive function, and the combination use with Chinese herbs as well as adverse effects.

## 2. Materials and Methods

### 2.1. Search Methods

The following electronic databases were searched up to July 2018: PubMed (in English), Medline (in English), Embase (in English), CNKI (in Chinese), WanFang DATA (in Chinese), and VIP (in Chinese). Search terms included “polycystic ovary syndrome /PCOS” and “berberine/BBR/Huang liansu (in Chinese)/Xiao bojian (in Chinese)”, and the research object was limited to only human beings. All studies that could possibly be related were preselected. The language was limited to both English and Chinese.

### 2.2. Selection and Exclusion Criteria

The selection criteria include randomized controlled trials (RCTs) and patients with PCOS-IR (PCOS diagnosed by the Rotterdam ESHRE/ASRM criteria, IR diagnosed by formula for insulin resistance revised by Hafner, 1996: homeostasis model assessment of insulin resistance (HOMA-IR) = [fasting blood glucose (FPG) (mmol/L) × fasting insulin (FIN) (mIU/L)]/22.5*⩾*1.66 [19]). The effect of BBR can be identified from the trials and the means and standard deviations (SD) of relevant indices before and after the trials were reported. Studies with the following characteristics were excluded: there was no other study with the same research design; the detailed data were not reported or were rated as low quality.

### 2.3. Data Collection and Analysis

The change of each index before and after treatment, which was calculated by subtracting baseline from final value, is the objective of the analyses in this study. The SD of the change (SD_change_) were calculated according to the formula offered by Cochrane Handbook for Systematic Reviews of Interventions Version 5.1.0 [20] (formula (1)) as they were not reported directly by the trials. Fixed effect model was used in indices without heterogeneity (I-squared<50%) and random effect model was used instead if apparent heterogeneity existed (I-squared>50%). Standardized Mean Difference (SMD) was used in measurement data [21]. The level of significance was set at P<0.05, and 95% confidence interval (95%CI) was given for all effect sizes. All data analysis in this study were conducted by Stata 13.0 software.

(1) SDchange=SDbaseline2+SDfinal2−2Corr×SDbaseline×SDfinal

**Notes:** Corr was set as 0.8 based on previous theory and experience for all the data analyses in this study.

## 3. Results

**Figure 1:**
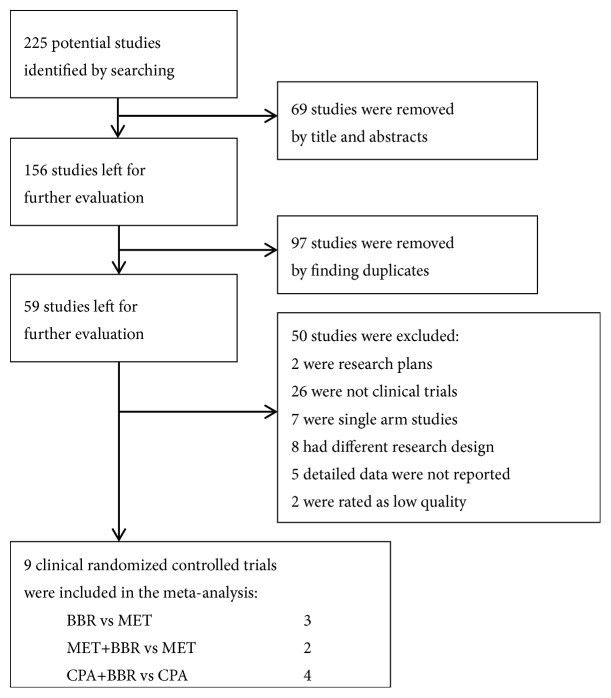
Flow diagram of the study screening process.** Notes:** BBR: berberine; MET: metformin; CPA: cyproterone acetate.

The screening process was shown as Figure 1. A total of 225 studies were identified by preliminary screening and 9 trials reported by 10 studies were taken into meta-analysis ultimately (Table 1). According to improved Jadad scale, the standards and scores of quality assessment for included trials are shown as Table 2.

### 3.1. BBR versus MET

A total of 3 trials (NO.1~NO.3) were processed for the effect of BBR in PCOS-IR treatment in contrast to MET. Meta-analysis showed no significant difference in the following items between BBR and MET group (Table 3). The forest plots are shown as Appendix 1. One-by-one eliminating study certified that the pooled effect is relatively reliable with low sensitivity and the value of Corr is appropriate (shown as Appendix 2).

### 3.2. MET+BBR versus MET

A total of 2 trials (NO.4 and NO.5) were included in the analysis to compare the effect of combination use of MET+BBR versus MET alone in the treatment of PCOS-IR. As is shown in Table 4, the MET +BBR group showed a more significant reduction in luteinizing hormone (LH), LH/follicle-stimulating hormone (FSH), and testosterone (T) than MET group (P<0.001). The forest plots are shown as Appendix 3.

### 3.3. CPA+BBR versus CPA

**Figure 2:**
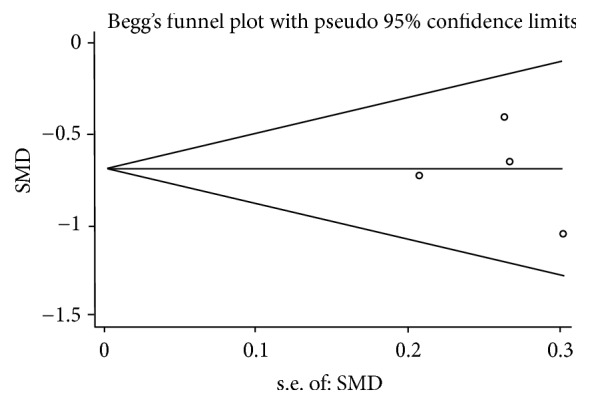
The Begg's funnel plot.

A total of 4 trials (No. 6~ No. 9) were included in the analysis to compare the effect of combination use of CPA+BBR versus CPA alone in the treatment of PCOS-IR. Significant difference was observed in waist hip rate (WHR), LH, FPG, FIN, HOMA-IR, total cholesterol (TC), triacylglycerol (TG), low-density lipoprotein cholesterol (LDL-C), and high-density lipoprotein cholesterol (HDL-C), which means when compared with MET group, CPA+BBR group showed a more significant reduction in WHR, LH, FPG, FIN, HOMA-IR, TC, TG, and LDL-C and a more significant increase in HDL-C (Table 5). The forest plots are shown as Appendix 4. The Begg's funnel plot (Figure 2) indicated that no publication bias existed (P=0.770).

## 4. Discussion

### 4.1. The Effect of BBR on Glycolipid Metabolism and Insulin Resistance

Obesity is a significant contributor clinical feature of some PCOS patients [22]. Body mass index (BMI) and WHR were positively correlated with insulin resistance [23]. Insulin resistance and compensatory hyperinsulinemia are the critical points of PCOS's pathogenesis. Dyslipidemia and insulin resistance are closely related [24]. The targets of PCOS treatment include reducing concentrations of androgens, increasing glycolipid metabolism, alleviating clinical symptoms such as obesity, hirsutism, acne, acanthosis, nigricans, and anovulation, improving the ovulation and pregnancy rate, and decreasing the long-term risk of endometrial cancer, diabetes, hypertension, and cardiovascular disease [12]. MET is a widely used insulin sensitizer and can effectively reduce the serum levels of insulin and androgen and regularize the cycle of menses. Our analysis shows that the within-group changes in BMI, HOMA-IR, TC, TC, TG, and LDL-C between last visit and baseline are significant in both BBR and MET group, whereas no significant difference existed in the between-group comparisons, which means that according to limited studies, BBR may have similar effect to MET in improving glycolipid metabolism and enhancing insulin sensitivity. Our analysis shows that the combination of MET and BBR is not superior to MET alone in the reduction of BMI and HOMI-IR, which conflicts with another report [16] and more RCTs are acquired. CPA is a first-line agent for PCOS for adjusting menstruation and reducing androgen [6]. Our analysis shows that the combination of CPA and BBR was more effective than CPA used alone in reducing WHR, HOMA-IR, TC, TG, and LDL-C and increasing HDL-C, which suggests the function of BBR when used in combination with CPA as an insulin sensitizer in improving carbohydrate metabolism and enhancing insulin sensitivity.

### 4.2. The Effect of BBR on Reproductive Endocrine Condition

Hyperandrogenism, together with the increase of LH and LH/FSH, is the main endocrine characteristics of PCOS. IR and hyperinsulinemia are recognized as the main inducements of hyperandrogenism and anovulation [25]. Insulin-sensitizing agents are commonly used as adjunct medication for women with PCOS to improve clinical symptoms by inhibiting insulin secretion [26]. Our analysis shows significant within-group changes of LH and T in BBR group but not in MET group, but no difference in the reduction of LH or T when comparing the two groups above. Between-group comparisons are significant between groups MET+BBR and MET alone in the reduction of LH, LH/FSH, and T. The reduction of LH in the combination group of CPA and BBR is superior to that of CPA alone, but the reduction of T was similar between the two groups above. In addition, Wei W. [16] reported that a significant increase in hormone-binding globulin (SHBG) was found in the between-group comparison of group CPA+BBR with CPA+MET or with CPA alone, and total testosterone (TT) had a significant decline after treatment in CPA+BBR group when compared with CPA alone. But the evidence is still insufficient to confirm whether the effect of MET+BBR is superior to MET alone, or CPA+BBR is superior to CPA alone in improving endocrine indices.

### 4.3. The Effect of BBR on the Ovulation, Pregnancy, and Live Birth Rate

Zhang A.P. [27] found in a single-arm study that the ovulation rate of PCOS patients increased from 24.44% to 65.56% (P<0.05) after BBR treatment (300 mg, tid, 3 months), and speculated that the result may be due to BBR's metabolic effects on improving insulin sensitivity. It has been indicated that MET, as a biguanide oral antihyperglycemic agent, may directly decrease ovarian androgen production [18]. Kuang H. [10] found that BBR (300 mg, tid, 3 months) was similar to MET (500 mg, bid, 3 months) in enhancing pregnancy rate. A similar RCT [13] showed no significant difference of pregnancy rate between BBR group (59.5%) and MET group (47.4%), whereas the live birth rate of BBR group (48.6%) is superior to MET group (36.8%). Wang L.X. [14] reported that the combination of MET and BBR had a higher ovulation rate than MET alone (76.13% versus 64.24%), and similar outcomes were found in the RCT performed by Wang P. [15] (83.33% versus 69.05%). It is speculated that BBR may induce ovulation, regularize menstruation, and enhance the rate of pregnancy and live birth by acting on hypothalamic-pituitary-ovarian axis (HPOA) [12]. Wu X.K. [28] found in one RCT enrolling 644 participants that the pregnancy and ovulation rates were similar in LET group (39% and 59%, respectively) and the combination group of LET and BBR (38% and 61%, respectively), and both rates were higher than those in the group of BBR alone. Meta-analysis of the ovulation, pregnancy, and live birth rates cannot be performed because they are reported by only a few RCTs with different research designs, which suggests that further studies are needed to evaluate the effect of BBR in improving reproductive function.

### 4.4. The Combination Use of BBR with Other Chinese Herbs in PCOS-IR Treatment

Recently, the effects of BBR in the combination with different kinds of Chinese herbs for PCOS-IR treatment have attracted a growing number of researchers' attention. Liu W. [29] and Wang Y.Y. [30] found out that BBR combined with Chinese prescription “Cang Fu Dao Tan Tang” reduced BMI, HOMA-IR, FIN, T, LH, and LH/FSH LDL-C, and the effect on BMI, HOMA-IR, LH, T, FIN, TG, LDL-C, and HDL-C of the combination group is superior to that of the “Cang Fu Dao Tan Tang” alone group. Cai L.H. [26] found that the ovulation rate showed significant difference between the experimental group of Chinese prescription “Gui Zhi Fu Ling Wan” + BBR and control group of CC +MET (87.78% and 73.33%, respectively) after 3 months of treatment, but difference was not found in pregnancy rate between the two groups (70.0% and 53.33%, respectively). Shao X.M. [31] reported that significant difference was found in the reduction of HOMA-IR, TG, HDL-C, LH, and LH/FSH between experimental group of “Gui Zhi Fu Ling Wan” + BBR and control group of CPA+MET, but the between-group comparisons of the outcomes of ovulation and pregnancy were not significant. Liu C.L. [32] found within-group changes of FIN, HOMA-IR, and TG in both group of Chinese prescription “You Gui Wan” + BBR and group CPA+MET between last visit and baseline, whereas the between-group comparison had no difference. Limited studies have shown that the combination use of BBR and Chinese herbs has positive effect on the treatment of PCOS-IR, but more well-designed trials should be conducted.

### 4.5. The Safety and Adverse Effects of BBR

The main adverse effects of MET, which is commonly used as an insulin-sensitizing agent for women with PCOS-IR, are stomach upset, loss of appetite [33], and kidney injury [34]. Kuang H. [10] reported 2 cases of diarrhea in BBR group as well as 7 cases of nausea or loss of appetite and 1 case of diarrhea in MET group. An Y. [12] reported that the incidence of nausea, vomiting, diarrhea, abdominal pain, and other adverse reactions in BBR group was lower than that in MET group. Zuo F. [35] found that the gastrointestinal adverse reaction in BBR group was less severe than that in MET group. But further studies are still needed to evaluate the adverse effects of BBR.

## 5. Conclusion

In summary, this meta-analysis and systemic review demonstrates a promising prospect of BBR in treating PCOS-IR, but the underlying mechanisms need further study. Considering the relatively small number of RCTs included in this meta-analysis and the sample size, more properly designed, randomized, double-blind, placebo-controlled trials are still needed to further confirm the effect and safety of BBR. Since BBR, MET, and CPA are different kinds of drugs which target different aspects of PCOS, the study of their mechanisms will benefit the exploration of the pathogenesis of PCOS.

## Figures and Tables

**Table 1:** The essential information of trials included in the meta-analysis.

**No.**	**Trials**	**Comparisons**	**Sample size**	**Protocol**	**Duration**	**Outcomes**
1	KUANG Heng (2014) [10]	BBR/MET	23/28	BBR:300mg tid/	3 months	BMI, WHR, FSH, LH, LH/FSH, T, HOMA-IR, TC, TG, HDL-C, LDL-C
				MET:500mg bid		

2	AN Yuan et al. (2014) (2016)[11, 12]	BBR/MET/P	44/41/43	BBR: 500mg tid/	3 months	BMI, WHR, FSH, LH, T, HOMA-IR, FBG, FIN, TC, TG, HDL-C, LDL-C
				MET: 500mg tid/		
				P: 1 tablet tid		

3	LI Xiaobin e-t al. (2017) [13]	BBR/MET	26/29	BBR:300mg tid/	3 months	BMI, HOMA-IR, FSH, LH, LH/FSH, T, FPG, FIN, TC, TG, HDL-C, LDL-C
				MET:500mg bid		

4	WANG Ling-xiao et al. (2011) [14]	MET+BBR/MET	28/28	MET:500mg tid +BBR:500mg tid /MET:500mg tid	3 months	BMI, LH, FSH, LH/FSH, T, FBS, FIN, HOMA-IR

5	WANG Ping et al(2016) [15]	MET+BBR/MET	42/42	MET:500mg tid +BBR:500mg tid /MET:500mg tid	3 months	BMI, WHR, FSH, LH, LH/FSH, HOMA-IR, T,

6	MA Yukun e-t al. (2011) [4]	CPA+BBR/CPA/CPA+BBR +MET/ CPA+MET	31/28/33/30	CPA:1 tablet qd+BBR:500mg bid/ CPA:1 tablet qd /	3 months	BMI, WHR, FPG, FIN, HOMA-IR, TC, TG, HDL-C, LDL-C, FSH, T, LH
				CPA:1 tablet qd +BBR:500mg bid+MET:500mg tid		
				CPA:1 tablet qd +MET:500mg tid		

7	WEI Wei et al.(2012) [16]	CPA+BBR/ CPA+MET/CPA +P	31/30/28	CPA 1 tablet qd+BBR:500mg tid /	3 months	BMI, WHR, FPG, FIN, HOMA-IR, TC, TG, HDL-C, LDL-C
				CPA 1 tablet qd +MET:500mg tid /		
				CPA 1 tablet qd + P:1 tablet bid		

8	ZHU Qiuyan et al. (2016) [17]	CPA+BBR/	25/25	CPA 1 tablet qd +BBR:300mg tid / CPA 1 tablet qd + P: 1 tablet tid	3 months	BMI, WHR, FPG, HOMA-IR, TC, TG FSH, LH, T
		CPA +P				

9	CHEN Xiao et al.(2016) [18]	CPA+BBR/	50/50	CPA 1 tablet qd +BBR:500mg bid / CPA 1 tablet qd	3 months	TC, TG, HDL-C, LDL-C, FPG, FIN
		CPA				

**Notes**: BBR: berberine; MET: metformin; CPA: cyproterone acetate; P: placebo; qd: once a day; bid: twice a day; tid: three times a day.

BMI: body mass index; FSH: follicle-stimulating hormone; LH: luteinizing hormone; T: testosterone; HOMA-IR: homeostasis model assessment of insulin resistance; TC: total cholesterol; TG: triacylglycerol; DLD-C: low-density lipoprotein cholesterol; HDL-C: high-density lipoprotein cholesterol; WHR: waist hip rate; FIN: fasting insulin; FPG: fasting blood glucose.

**Table 2:** Quality assessment of included trials.

**No.**	**Trials**	**Random allocation**	**Allocation concealment**	**Blinding**	**Loss to follow-up**	**Jadad score**
1	KUANG Heng (2014)	2	1	1	1	5

2	AN Yuan et al. (2014)(2016)	1	1	2	1	5

3	LI Xiaobin et al. (2017)	2	1	1	1	5

4	WANG Lingxiao et al. (2011)	1	1	1	1	4

5	WANG Ping et al. (2016)	1	1	1	1	4

6	MA Yukun et al. (2011)	1	1	1	1	4

7	WEI Wei et al. (2012)	2	1	2	1	6

8	ZHU Qiuyan et al. (2016)	2	1	2	1	6

9	CHEN Xiao et al. (2016)	2	1	1	1	5

**Notes:** The standards for evaluation:

**Random allocation:** 2: appropriate random allocation was used; 1: random allocation was used but the method was unclear; 0: random allocation was inappropriately used or was not used.

**Allocation concealment:** 2: appropriate allocation concealment was used; 1: allocation concealment was used but the method was unclear; 0: the allocation concealment was inappropriately used or was not used.

**Blinding:** 2: appropriate blinding was used; 1: blinding was used but the method was unclear; 0: the blinding was inappropriately used or was not used.

**Loss to follow-up:** 1: the number and reason of loss to follow-up were described; 0: the number and reason of loss to follow-up were not described.

**Jadad score:** 1-3: low quality; 4-7: high quality.

**Table 3:** The result of meta-analysis regarding the effect of BBR versus MET.

**Indices**	**SMD**	**95**%**CI**	**P**	**Trials involved**
BMI^◆1♣1◆2♣2◆3♣3^	-0.158^*◤*☆^	-0.446~1.130	0.281^*◎*^	No. 1, No. 2, No. 3

FSH	0.184^*◥*^	-0.305~0.673	0.461^*◎*^	No. 1, No. 2, No. 3

LH^◆1◆3^	-0.130^*◥*☆^	-0.688~0.429	0.649^*◎*^	No. 1, No. 2, No. 3

T^◆1◆2♣2◆3^	-0.516^*◥*☆^	-1.088~0.055	0.077^*◎*^	No. 1, No. 2, No. 3

HOMA-IR^◆1♣1◆2♣2◆3^	-0.188^*◤*☆^	-0.476~0.100	0.201^*◎*^	No. 1, No. 2, No. 3

TC^◆1♣1◆2♣2◆3♣3^	-1.233^*◥*☆^	-2.912~0.446	0.150^*◎*^	No. 1, No. 2, No. 3

TG^◆1♣1◆3♣3^	0.045^*◤*^	-0.243~0.332	0.761^*◎*^	No. 1, No. 2, No. 3

LDL-C^◆1♣1◆2♣2◆3♣3^	-0.701^*◥*☆^	-1.630~0.229	0.140^*◎*^	No. 1, No. 2, No. 3

HDL-C	0.148^*◥*^	-0.984~1.280	0.798^*◎*^	No. 1, No. 2, No. 3

Notes:

^*◆*x^: significant statistical difference (P<0.05) between final value and baseline in BBR group was reported by trial No. x.

^*♣*x^: significant statistical difference (P<0.05) between final value and baseline in MET group was reported by trial No. x.

^*◤*^:fixed effect model;^*◥*^:random effect model.

^*◎*^: P>0.05.

^☆^: BBR group showed a greater change than MET before and after treatment.

**Table 4:** The result of meta-analysis regarding the effect of MET+BBR versus MET.

**Indices**	**SMD**	**95**%**CI**	**P**	**Trials involved**
BMI^◆4♣4^	-0.670^*◥*☆^	-1.927~0.587	0.296^*◎*^	No. 4, No. 5

FSH	-0.025^*◤*☆^	-0.357~0.306	0.880^*◎*^	No. 4, No. 5

LH^◆4♣4^	-0.663^*◤*☆^	-0.974~-0.293	0.001*∗∗∗*	No. 4, No. 5

LH/FSH^◆4♣4^	-0.763^*◤*☆^	-1.108~-0.419	0.001*∗∗∗*	No. 4, No. 5

T^◆4♣4^	-0.629^*◤*☆^	-0.969~-0.290	0.001*∗∗∗*	No. 4, No. 5

HOMA-IR^◆4♣4^	-1.113^*◥*☆^	-2.516~0.289	0.120^*◎*^	No. 4, No. 5

Notes:

^*◆*x^: significant statistical difference (P<0.05) between final value and baseline in MET+BBR group was reported by trial No. x.

^*♣*x^: significant statistical difference (P<0.05) between final value and baseline in MET group was reported by trial No. x.

Statistical difference between final value and baseline in each group was not reported in trial No. 5.

^*◤*^:fixed effect model;^*◥*^:random effect model.

^*◎*^: P>0.05; *∗∗∗*: P<0.001.

^☆^: MET+BBR group showed a greater change than MET before and after treatment.

**Table 5:** The result of meta-analysis regarding the effectiveness of CPA+BBR versus CPA.

**Indices**	**SMD**	**95**%**CI**	**P**	**Trials involved**
BMI^◆6♣6◆7♣7^	-0.235^*◥*☆^	-0.681~0.211	0.302^*◎*^	No. 6, No. 7, No. 8

WHR^◆6♣6◆7♣7^	-0.942^*◥*☆^	-1.755~-0.129	0.023*∗*	No. 6, No. 7, No. 8

FSH	2.807^*◥*^	-2.688~8.301	0.317^*◎*^	No. 6, No. 8

LH^◆6^	-0.723^*◤*☆^	-1.111~-0.335	0.001*∗∗∗*	No. 6, No. 8

T^◆6♣6^	-0.484^*◥*☆^	-1.062~0.093	0.100^*◎*^	No. 6, No. 8

FPG^◆6◆7^	-0.688^*◤*☆^	-0.936~-0.441	0.001*∗∗∗*	No. 6, No. 7, No. 8, No. 9

FIN^◆6◆7♣7^	-0.620^*◤*☆^	-0.893~-0.348	0.001*∗∗∗*	No. 6, No. 7, No. 9

HOMA-IR^◆6◆7♣7^	-0.713^*◤*☆^	-1.026~-0.400	0.001*∗∗∗*	No. 6, No. 7, No. 8

TC^◆6◆7♣7^	-3.816^*◥*☆^	-6.188~-1.444	0.002*∗∗*	No. 6, No. 7, No. 8, No. 9

TG^◆6◆7♣7^	-1.516^*◥*☆^	-2.112~-0.920	0.001*∗∗∗*	No. 6, No. 7, No. 8, No. 9

LDL-C^◆6◆7^	-1.173^*◥*☆^	-1.661~-0.685	0.001*∗∗∗*	No. 6, No. 7, No. 9

HDL-C^◆6◆7^	1.452^*◤*^	1.152~1.752	0.001*∗∗∗*	No. 6, No. 7, No. 9

Notes:

^*◆*x^: significant statistical difference (P<0.05) between final value and baseline in CPA+BBR group was reported by trial No. x.

^*♣*x^: significant statistical difference (P<0.05) between final value and baseline in CPA group was reported by trial No. x.

Statistical difference between final value and baseline in each group was not reported in trial No. 8 and 9.

^*◤*^:fixed effect model;^*◥*^:random effect model.

^*◎*^: P>0.05; *∗*: P<0.05; *∗∗*: P<0.01; *∗∗∗*: P<0.001.

^☆^: CPA+BBR group showed a greater change than CPA before and after treatment.

## Supplementary Materials

Supplementary Materials Appendix 1. Forest Plots for BBR versus MET. Appendix 2. Results of Sensitivity Analyses for BBR versus MET. Appendix 3. Forest Plots for MET+BBR versus MET. Appendix 4. Forest Plots for CPA+BBR versus CPA (continued).
