# Supplementary material for: The Effect of Berberine on Polycystic Ovary Syndrome Patients with Insulin Resistance (PCOS-IR): A Meta-Analysis and Systematic Review
Source: Evid Based Complement Alternat Med. 2018 Nov 14;2018:2532935. doi: 10.1155/2018/2532935 (PMC6261244; doi:10.1155/2018/2532935)

APPENDIXES

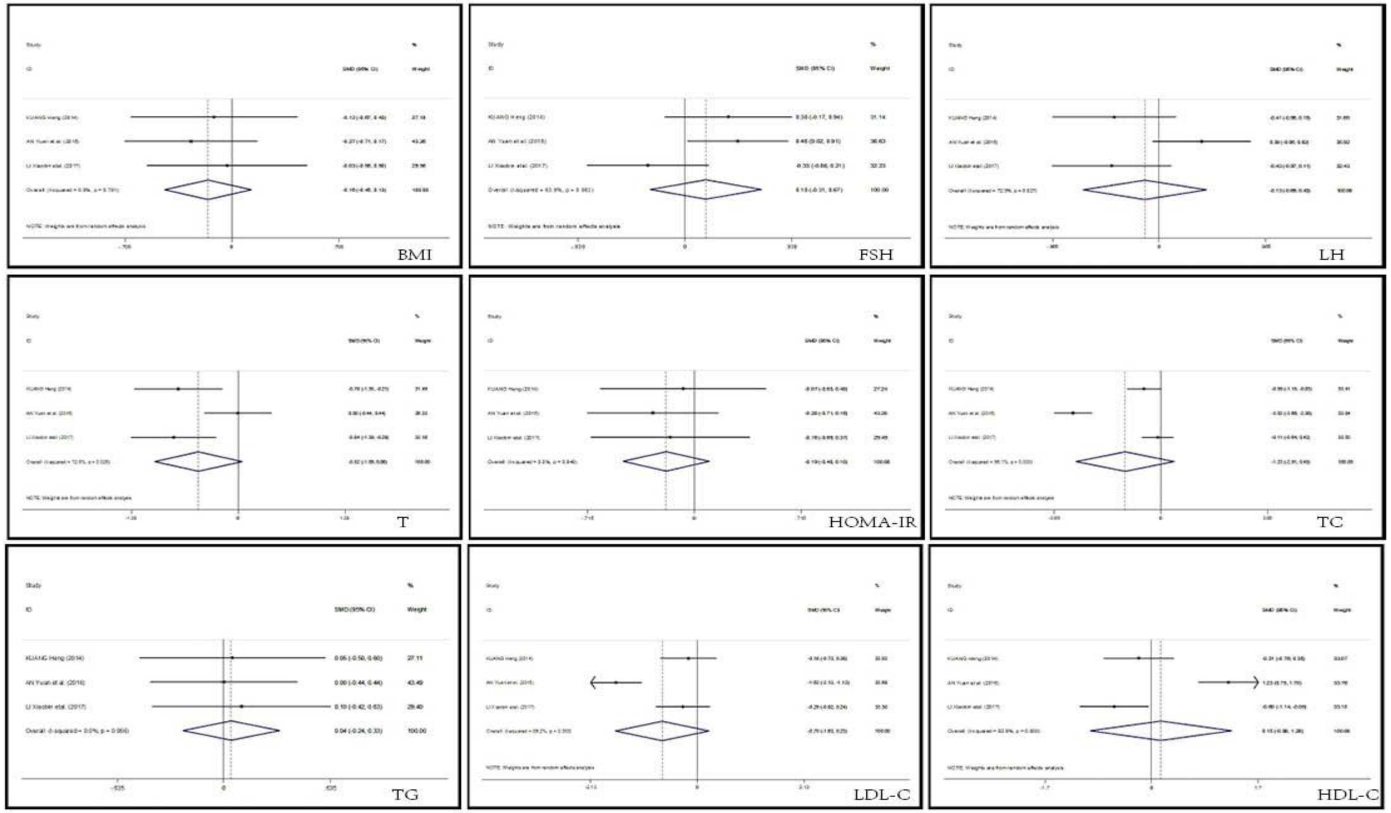

Appendix 1. Forest Plots for BBR vs. MET

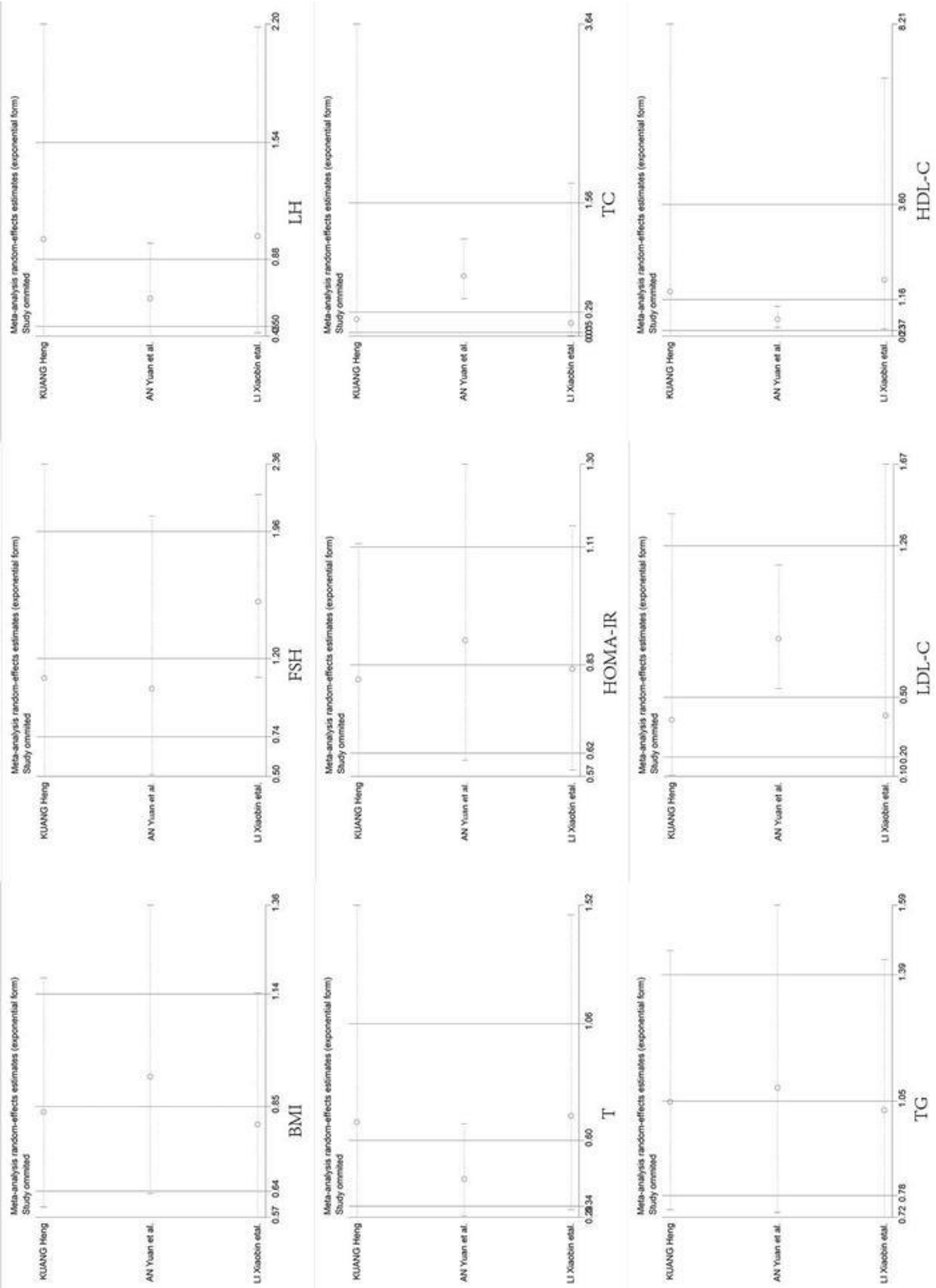

Appendix 2. Results of Sensitivity Analyses for BBR vs. MET

Appendix 3. Forest Plots for MET+BBR vs. MET

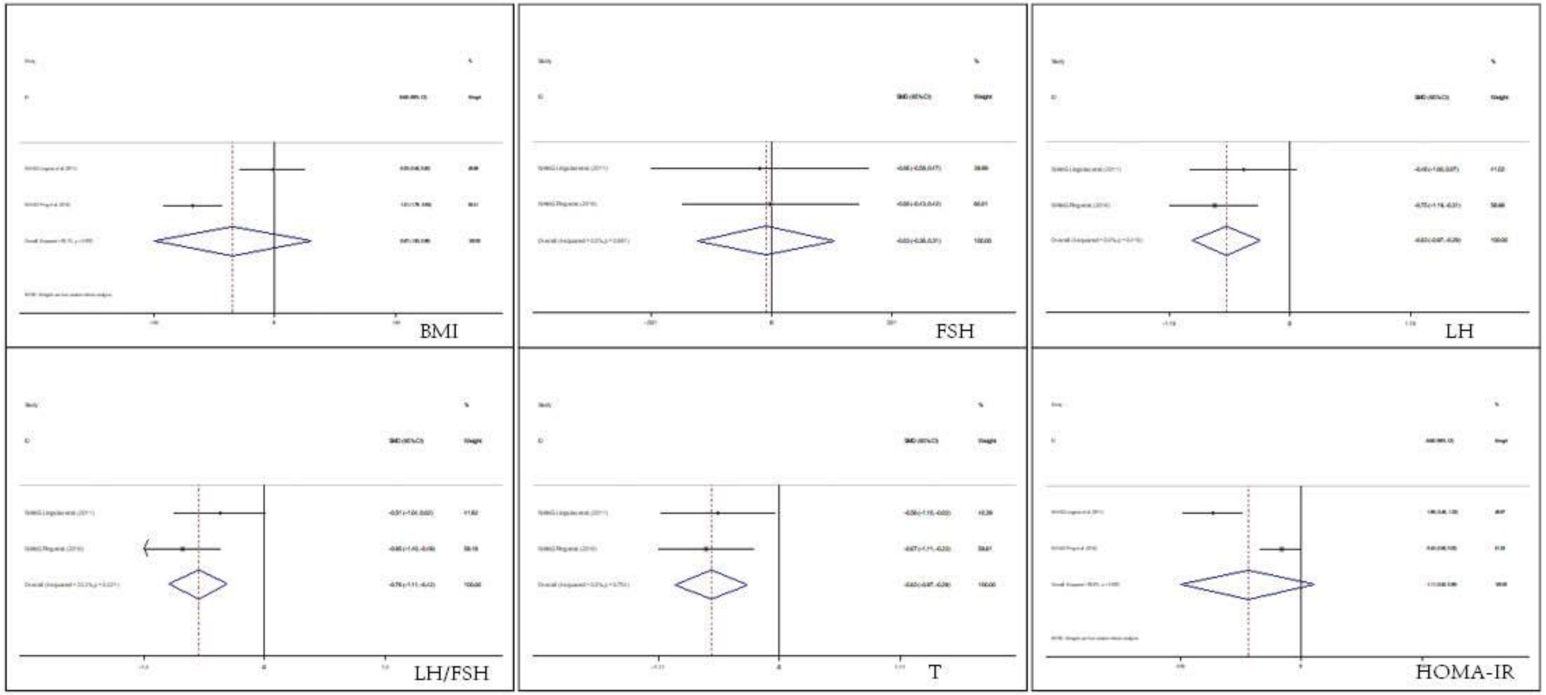

Appendix 4. Forest Plots for CPA+BBR vs. CPA

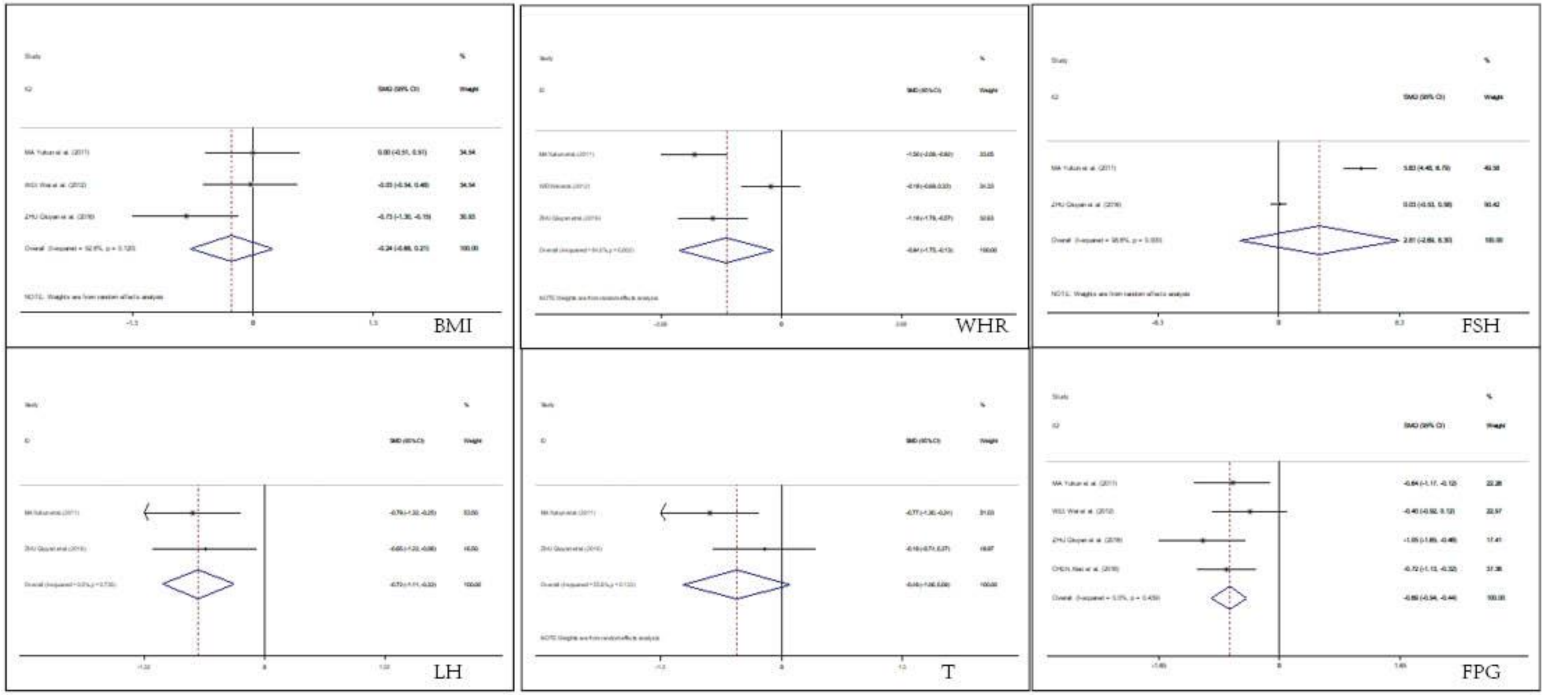

Appendix 4. Forest Plots for CPA+BBR vs. CPA（continued）

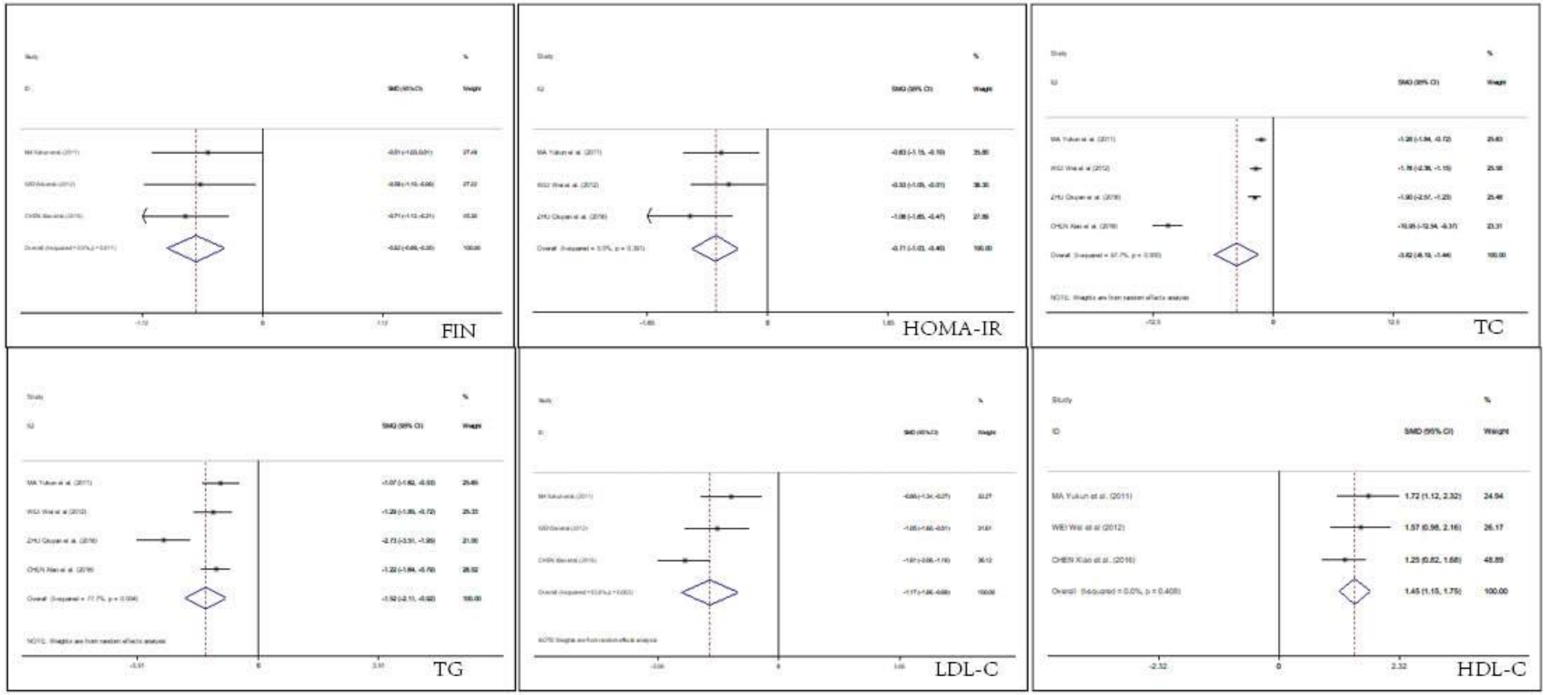

Supplement: Supplementary Materials — Appendix 1. Forest Plots for BBR versus MET. Appendix 2. Results of Sensitivity Analyses for BBR versus MET. Appendix 3. Forest Plots for MET+BBR versus MET. Appendix 4. Forest Plots for CPA+BBR versus CPA (continued). [file 2532935.f1.pdf]
